# Comparative RNA function analysis reveals high functional similarity between distantly related bacterial 16 S rRNAs

**DOI:** 10.1038/s41598-017-10214-3

**Published:** 2017-08-30

**Authors:** Miyuki Tsukuda, Kei Kitahara, Kentaro Miyazaki

**Affiliations:** 1 Department of Life Science and Biotechnology, Bioproduction Research Institute, National Institute of Advanced Industrial Science and Technology (AIST), Tsukuba, Ibaraki 305-8566 Japan; 2 Department of Computational Biology and Medical Sciences, Graduate School of Frontier Sciences, The University of Tokyo, Kashiwa, Chiba 277-8561 Japan; 3 Ambitious Leader’s Program, Graduate School of Science, Hokkaido University, N10W8, Kita-ku, Sapporo 060-0810 Japan

## Abstract

The 16 S rRNA sequence has long been used uncritically as a molecular clock to infer phylogenetic relationships among prokaryotes without fully elucidating the evolutionary changes that this molecule undergoes. In this study, we investigated the functional evolvability of 16 S rRNA, using comparative RNA function analyses between the 16 S rRNAs of *Escherichia coli* (Proteobacteria) and Acidobacteria (78% identity, 334 nucleotide differences) in the common genetic background of *E. coli*. While the growth phenotype of an *E. coli* mutant harboring the acidobacterial gene was disrupted significantly, it was restored almost completely following introduction of a 16 S rRNA sequence with a single base-pair variation in helix 44; the remaining 332 nucleotides were thus functionally similar to those of *E. coli*. Our results suggest that 16 S rRNAs share an inflexible cradle structure formed by ribosomal proteins and have evolved by accumulating species-specific yet functionally similar mutations. While this experimental evidence suggests the neutral evolvability of 16 S rRNA genes and hence satisfies the necessary requirements to use the sequence as a molecular clock, it also implies the promiscuous nature of the 16 S rRNA gene, i.e., the occurrence of horizontal gene transfer among bacteria.

## Introduction

The ribosome is an RNA-centered and highly complex ribonucleoprotein particle, in which mRNA-encoded genetic information is translated into proteins^1, 2^. As this biological process is essential to sustain cellular activities, the ribosome is found in every type of cellular organism, including bacteria, archaea, and eukaryotes.

Carl Woese first proposed that the systematic comparison of small subunit ribosomal RNA (SSU rRNA: 16 S rRNA for prokaryotes and 18 S rRNA for eukaryotes) sequences from different organisms would make it possible to infer the evolutionary relationships among organisms in the form of phylogenetic trees^3–5^. Recursive phylogenetic mapping of each newly discovered (micro-) organism onto an already constructed phylogenetic tree has progressively established a persuasive global tree of life^4, 6–9^, and this pictorial concept has penetrated deeply into the field of biology as a consensus view on the way organisms have evolved^3^. Having stood the test of time for over 30 years, this SSU rRNA sequence-based approach remains the gold standard method to infer organismal phylogeny.

However, considering the fact that decisive fossil records, by which any molecular clock should be validated or calibrated, are not available for prokaryotes, strong theoretical basis such as the existence of neutral evolvability or excluding the possibility of horizontal gene transfer (HGT) events should then be assured before a specific gene is used as a molecular clock. Currently, we lack evidence for both of these aspects and therefore cannot answer simple questions such as whether 16 S rRNA has evolved neutrally (without interacting with ribosomal proteins) or whether the complexity hypothesis^10^ applies to 16 S rRNA, which is a representative “informational gene” product, to prevent HGT within the context of the highly complex ribosomal particle^1, 2^ of each organism. To address the molecular evolvability of 16 S rRNA, we propose a new methodology named the comparative RNA function (CRF) analysis, in which the functional similarity or dissimilarity of 16 S rRNAs are analyzed experimentally, exclusively focusing on the functional effects of mutations that accumulate differently in respective rRNAs during speciation.

In this study, using the *Escherichia coli* ribosomal system as a common platform, we conducted a CRF analysis on the 16 S rRNAs between *E. coli* and Acidobacteria, which are phylogenetically distinct at the phylum level, with only 78% identity (or 334 nucleotide differences out of a total of ~1,500 nucleotides). Our experimental results revealed that 332 (99.4%) nucleotides in the acidobacterial 16 S rRNA gene were functionally similar to those in *E. coli* genetic background, providing strong evidence that the primitive 16 S rRNAs were held by a common framework of ribosomal proteins and then accumulated lineage-specific neutral mutations during evolution. While this functional similarity in distantly related 16 S rRNAs seemingly assures the use of these sequences as a reliable clock, it also suggests a unique evolutionary characteristic of the gene; 16 S rRNA is quite amenable to HGT, highlighting the promiscuous nature of the gene.

## Results

### Functional compatibility of 16 S rRNA across bacterial phyla

To obtain 16 S rRNAs that are evolutionary distinct yet functional in *E. coli*, we screened a metagenomic library of 16 S rRNA genes in the *E. coli* strain KT105 (the null mutant of all seven *rrn* operons in the genome), following a previously described procedure^11^ with modifications, as described in the Supplementary Materials and Methods. We identified a clone of the 16 S rRNA gene from an acidobacterial species (designated 16S^NS11^) showing a minimal sequence identity of 78.4% to that of *E. coli* (16S^Eco^) (Supplementary Table 1). We performed a CRF analysis using 16S^Eco^ and 16S^NS11^ to investigate how these sequence differences affect the function of 16 S rRNA.

**Figure 1:**
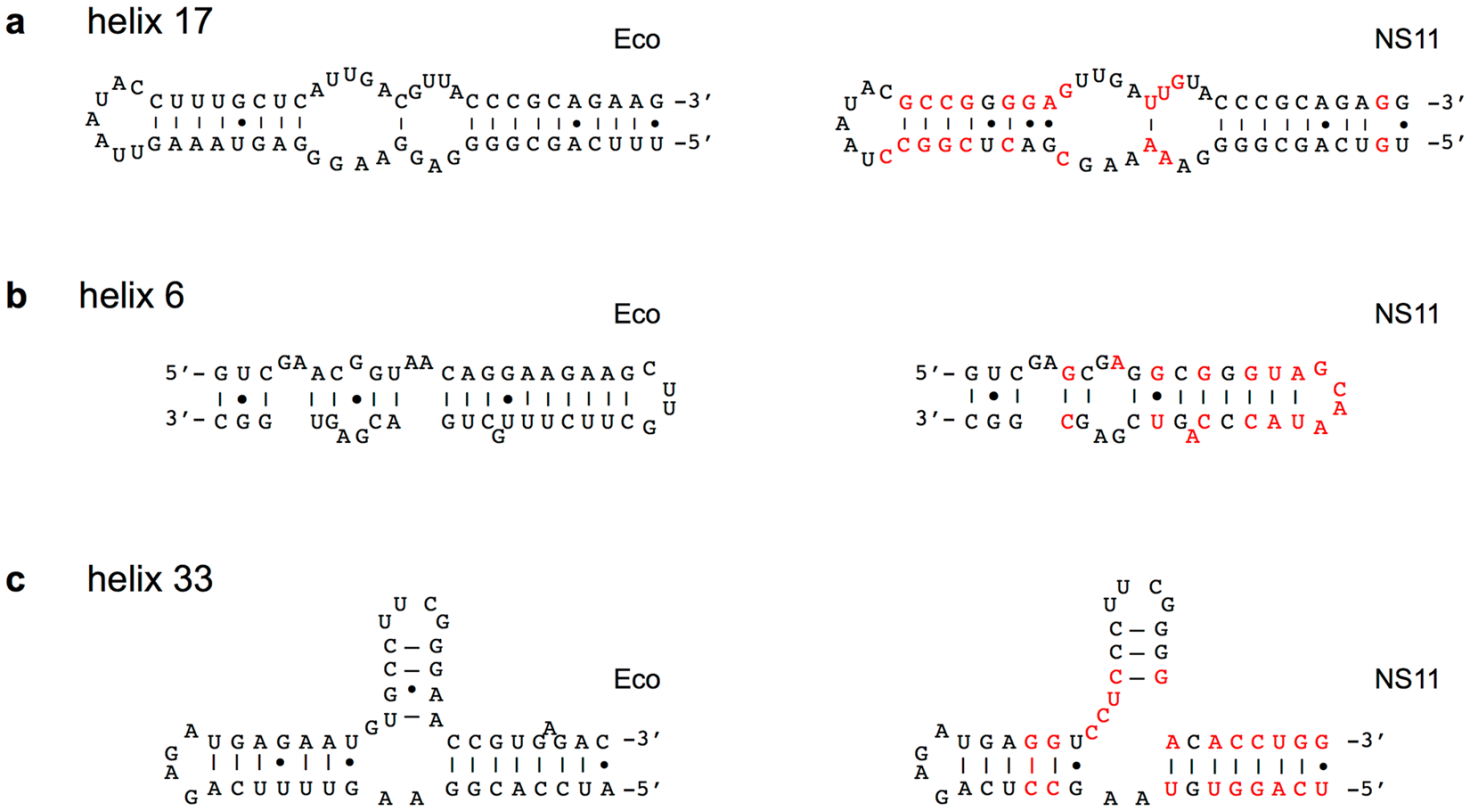
Comparison of the secondary structures of *E. coli* and acidobacterial 16 S rRNAs. The secondary structures of the 16 S rRNA sequences of *E*. *coli* (Eco) and an acidobacterial clone (NS11) are shown: (**a**) helix 17, (**b**) helix 6, and (**c**) helix 33. The NS11 16 S rRNA, which was shown to be functional in *E*. *coli*, shows 78.4% sequence identity (334 nucleotide differences) to Eco 16 S rRNA. Nucleotides that differ between the Eco and NS11 sequences are shown in red in the NS11 structure. For the complete secondary structures, see Supplementary Fig. 1.

The 16S^NS11^ differed from 16S^Eco^ by 334 nucleotides (Supplementary Fig. 1), among which 88 nucleotides had direct interactions with ribosomal proteins (dotted in red in Supplementary Fig. 2A). Overall, the secondary structure of the RNA helices appeared to be preserved between 16S^Eco^ and 16S^NS11^ according to the compensatory mechanism as shown in the RNA–RNA contact map in Supplementary Fig. 2B ^12, 13^. A typical example is shown in Fig. 1a for the helix 17 (h17) structure. However, we also found larger sequence and structural differences in some of the helices. For example, helix 6 (h6) differed between 16S^Eco^ and 16S^NS11^ in both the bulge and stem structures (Fig. 1b). In helix 33 (h33), several nucleotides were inserted in 16S^NS11^ (Fig. 1c). Therefore, not only are the typical compensatory mutations distinct between the two sequences but also the several unpaired (insertion/deletion) mutations.

### The 3′ minor domain of the acidobacterial 16 S rRNA deleteriously affects *E. coli* growth

**Figure 2:**
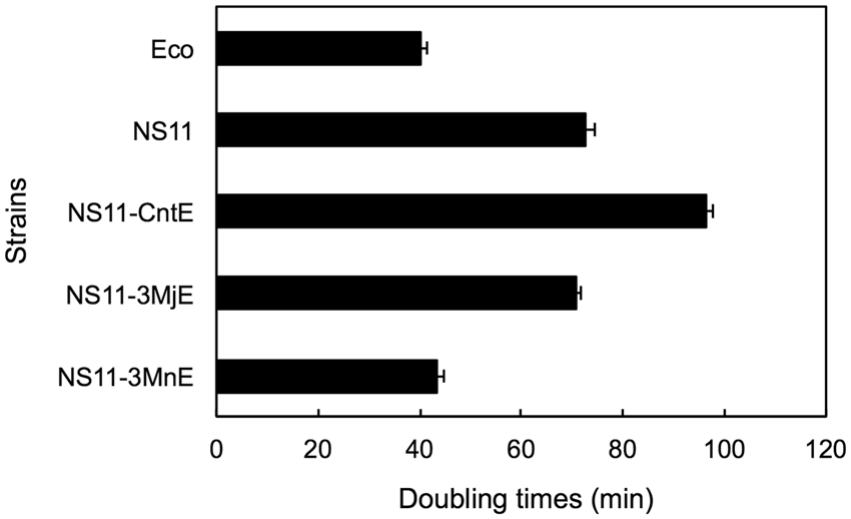
Disrupted function of acidobacterial 16 S rRNA in *E*. *coli* is significantly restored following replacement of the 3′ minor domain sequence with that of *E*. *coli* 16 S rRNA. The doubling times (DTs) of *E*. *coli* KT105 strains carrying 16 S rRNA sequences of *E*. *coli* (Eco), Acidobacteria (NS11), and their chimeras (NS11-CntE, NS11-3MjE, and NS11-3MnE) are shown. The 16 S rRNAs of NS11-CntE, NS11-3MjE, and NS11-3MnE have chimeric sequences based on NS11 16 S rRNA, in which the central domain, 3′ major domain, and 3′ minor domain sequences were replaced with the corresponding sequences of Eco 16 S rRNA. For the detailed construction of these chimeric variants, see Supplementary Fig. 3. All the strains were grown in LB medium at 37 °C. The DTs were the average of four independent experiments (*error bars*, SD). The KT105 strain carrying the NS11-5E 16 S rRNA was excluded from the graph due to its lack of viability (see text).

We measured the doubling times (DTs) of the *E. coli* KT105 strains containing 16S^Eco^ and 16S^NS11^ (Fig. 2). We found that KT105 with 16S^NS11^ (KT105/16S^NS11^) showed a significantly increased DT (72.6 min), indicating decreased viability compared to that of KT105/16S^Eco^ (40.2 min). Thus, introduction of a foreign 16 S rRNA sequence perturbed the function of the ribosome and decreased the host’s viability. There are two possible reasons for the growth perturbation. One possibility is that the foreign 16 S rRNA (16S^NS11^ in this case) contained a number of nucleotides that differ from those of the host 16S rRNA sequence (16S^Eco^ in this case), each of which would be slightly deleterious but not lethal. Such slightly deleterious sites would cumulatively cause significant perturbation of ribosomal functionality. In this situation, the viability of the host should be inversely correlated to the number of deleterious sites; step-by-step back mutation to the *E. coli* sequence would gradually recover the host’s viability. The second possibility is that the deleterious nucleotide(s) of the foreign 16 S rRNA (16S^NS11^ in this case) localizes in a specific region, and the remaining nucleotides that differ are functionally neutral. If so, the growth of the host (*E. coli* in this case) would be restored rapidly by a relatively small number of back mutations.

To identify the nucleotide(s) responsible for the perturbation of growth in KT105/16S^NS11^, we designed a series of chimeric 16 S rRNAs of 16S^Eco^ and 16S^NS11^, in which one of the four domains (5′, central, 3′ major, and 3′ minor) in 16S^NS11^ was replaced with the corresponding domain of 16S^Eco^. The resulting chimeric genes (16S^NS11-5E^, 16S^NS11-CntE^, 16S^NS11-3MjE^, and 16S^NS11-3MnE^) (Supplementary Fig. 3) were each introduced into *E. coli* KT101 to construct a chimeric mutant series of KT105. We found that 16S^NS11-CntE^, 16S^NS11-3MjE^, and 16S^NS11-3MnE^ supported the growth (colony formation) of KT105, whereas 16S^NS11-5E^ did not. The loss of the functionality in 16S^NS11-5E^ was presumably due to the disruption of the central pseudoknot structure (h2), which is formed between the 5′ major and central domains and is essential for ribosomal function^14–16^. This disruption was technically unavoidable in designing the 16S^NS11-5E^ chimera.

Next, DTs were determined for the viable chimeric mutants (i.e., KT105/16S^NS11-CntE^, KT105/16S^NS11-3MjE^, and KT105/16S^NS11-3MnE^) (Fig. 2). Prolonged DT was observed for KT105/16S^NS11-CntE^ (96.6 min), suggesting that partial disruption of the functional interactions between the 5′ and the central domains caused defective growth, though not complete lethality as observed for KT105/16S^NS11-5E^. No obvious recovery in DT was observed for KT105/16S^NS11-3MjE^ (70.9 min), suggesting that 3′ major domains of *E. coli* and NS11 are functionally similar. In contrast, KT105/16S^NS11-3MnE^ showed a significant recovery of viability; DT was shortened from 72.6 min to 43.6 min, which was similar to KT105/16S^Eco^ (40.2 min). Therefore, it was strongly suggested that the deleterious nature of 16S^NS11^ in the *E. coli* ribosomal particle was predominantly due to the 3′ minor domain. As the 3′ minor domain is the smallest among the four domains (~150 bases, whereas the entire 16S^Eco^ sequence is 1542 bases), the first possibility mentioned above could be ruled out. In contrast, the second possibility—that localization of deleterious sites in a specific region (the 3′minor domain in the present case)—seemed plausible.

### Biochemical characterization of mutant ribosomes

To confirm that the defective growth of the 16S rRNA-substituted strains was due to decreased ribosomal function, we conducted biochemical characterization of the ribosomes. *In vitro* translational activity of the ribosomes was determined using the *E. coli* cell-free transcription/translation system. The 70 S ribosome was purified from each KT105 mutant strain, and the activity was measured using green fluorescent protein (GFP)^17, 18^ as a reporter. The reaction was initiated by the addition of purified 70 S ribosome into the assay solution, and the increase of GFP fluorescence was measured over 5 h. As shown in Supplementary Fig. 4A, the activity of the ribosome with 16S^NS11^ was approximately a third of that of the ribosome with 16S^Eco^, which was markedly restored in the ribosome with 16S^NS11-3MnE^. Further characterization of the ribosomes was performed using sucrose density gradient analysis to study their subunit composition (Supplementary Fig. 4B). The mutated ribosome with 16S^NS11^ displayed a decreased assembly ratio of the 30 S subunit and association ratio between 30 S and 50 S subunits, both of which were markedly restored by substituting the 3′ minor domain with that of *E. coli* (16S^NS11-3MnE^). These results confirmed that the growth phenotype and ribosomal functions were well correlated.

### 99.4% of sequence variations between Acidobacteria and *E. coli* 16S rRNA are functionally similar with each other

**Figure 3:**
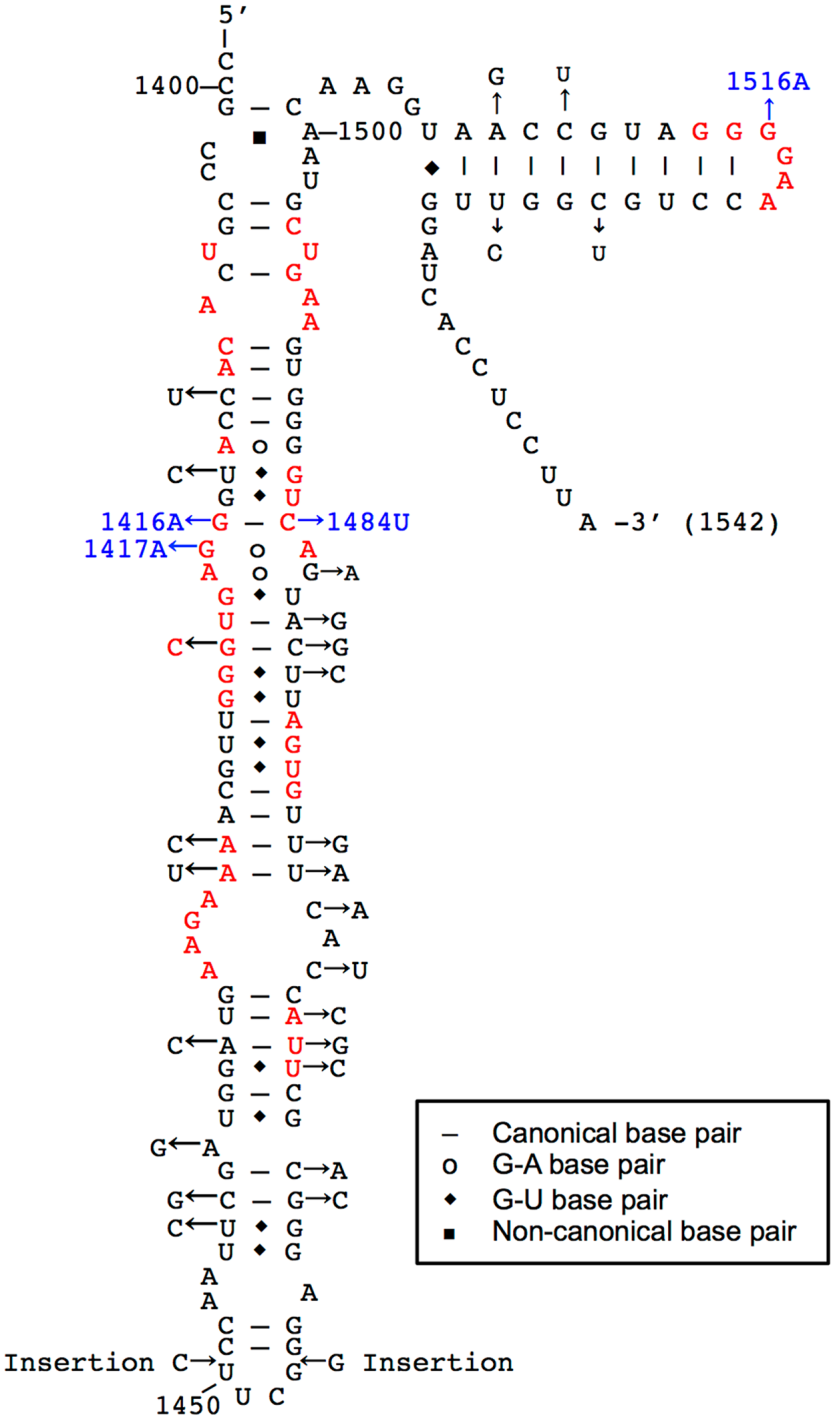
Sequence variation between the 16 S rRNA 3′ minor domains of *E*. *coli* and Acidobacteria. A secondary structure map of the 3′ minor domain of *E*. *coli* (Eco) 16 S rRNA is shown. Functionally important nucleotides known to be involved in subunit assembly and association are indicated in red (see text for details). Nucleotide differences between acidobacterial (NS11) and Eco 16 S rRNA are indicated by arrows, with the nucleotides beside the arrows. The 3′ end nucleotide (A1542) is also indicated.

According to the above-mentioned second possibility (presence of deleterious sites in the 3′ minor domain), we continued to identify the deleterious nucleotide(s) within the domain of 16S^NS11^. Figure 3 shows the secondary structure map of the 3′ minor domain of 16S^Eco^. Forty-one functionally important nucleotides for subunit-subunit interaction and ribosome biogenesis reported in the literature are shown in red^19–23^. Thirty-two nucleotides, which differ between 16S^Eco^ and 16S^NS11^, are indicated by arrows, and the corresponding nucleotides for 16S^NS11^ are indicated beside each arrow. Among these 32 nucleotides, we narrowed down the list of potentially deleterious nucleotides to ten (nucleotides 1416, 1417, 1421, 1429, 1430, 1463, 1464, 1465, 1484, and 1516) considering their functional importance. Of these, nucleotides 1463, 1464, and 1465 are located in the highly variable region of h44^24^, and nucleotides 1429 and 1430 in 16S^Eco^ do not affect the host’s growth upon mutation^25^. Nucleotide 1421 is involved in the central helix of h44, which pairs with nucleotide 1479. In 16S^Eco^, this region is G1421–C1479, whereas in 16S^NS11^, the base pair was replaced with C1421–G1479. Regarding the secondary structure, it was shown previously that the conservation of base pairing is critical, whereas the base identity is insignificant^26^. Thus, we excluded these six nucleotides from the subsequent analysis and focused on the following four sites: nucleotides 1416, 1417, 1484, and 1516 (shown in blue in Fig. 3). In 16S^Eco^, G1416 and G1417 are involved in the assembly of the 30 S subunit^27^, whereas C1484 and G1516 contribute to the interaction between 30 S and 50 S subunits involved in bridge B3 and bridge B2b, respectively^21^. One of the 11 post-transcriptionally modified bases is G1516, which is methylated by the site-specific methyltransferase RsmJ (encoded by the *ygiQ* gene)^28^.

We introduced mutations at four positions (A1416G, A1417G, U1484C, and A1516G), causing each of the four nucleotides in 16S^NS11^ to revert to the respective 16S^Eco^ sequence (Fig. 3). In addition, since nucleotides 1416 and 1484 form a base pair, we created the double mutant A1416G/U1484C. These variant genes were generated by site-directed mutagenesis using 16S^NS11^ as a template and then introduced into the *E. coli* host. Among the mutants, the A1416G mutant did not support growth (colony formation); however, the mutation likely retained the non-Watson-Crick base pair (G1416–U1484) (Fig. 3). The same base pair (G1416–U1484) in 16S^Eco^ was reported to allow growth, although the growth rate was reduced remarkably^25^. The reason for this discrepancy remains unclear, but it might be due to the difference in 16 S rRNA background (16S^Eco^ or 16S^NS11^). The U1484C mutation in 16S^NS11^ should result in disruption of the base pair (A1416–U1484 to A1416–C1484), but the resulting mutant retained viability. Sun *et al*. suggested that the mutation involved in bridge B3 might affect the conformational rearrangements of the 30 S initiation complex, which is required for association with the 50 S subunit and/or the ordered formation of bridging interactions during subunit association^25^. Considering the fact that the base pair-disrupting mutation in 16S^NS11^ was not detrimental and that the C1484G mutation in 16S^Eco^ retained functionality^25^, base-pairing between nucleotides 1416 and 1484 may not be essential for its function.

**Figure 4:**
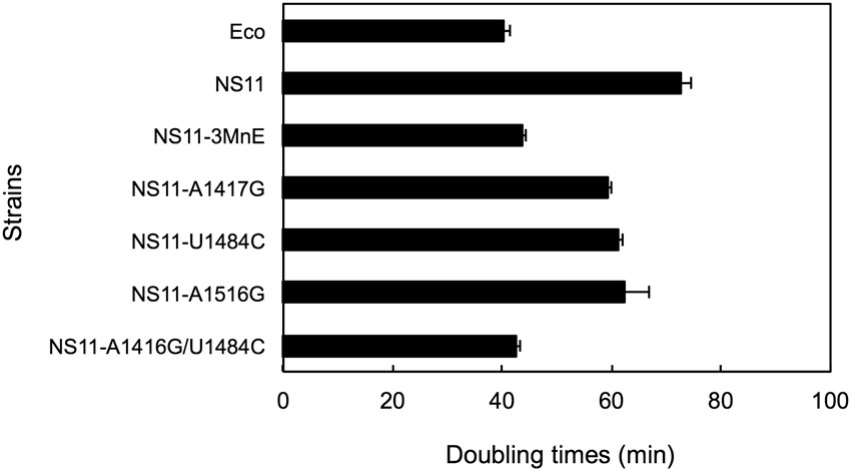
Acidobacterial 16 S rRNA is predominantly functionally neutral in *E*. *coli* except for base pairs 1416–1484. The doubling times (DTs) of *E*. *coli* KT105 strains carrying acidobacterial (NS11) 16 S rRNA sequences with point mutations in the 3′ minor domain (NS11-A1417G, NS11-U1484C, NS11-A1516G, and NS11-A1416/U1484C) are shown, accompanied by the DTs of KT105 strains carrying *E*. *coli* (Eco), NS11 (without point mutations), and NS11-3MnE 16 S rRNA sequences as references (same as Fig. 2). All the strains were grown in LB medium at 37 °C. The DTs were the average of four independent experiments (*error bars*, SD).

Next, DTs were determined for the viable point mutants. Overall, all functional single mutants showed better growth rates than the parental *E. coli* KT105/16S^NS11^ (Fig. 4). In particular, the DTs of the double mutant KT105/16S^NS11-A1416G/U1484C^ were drastically shortened from 72.6 min (KT105/16S^NS11^) to 42.7 min, which was slightly shorter than that of the chimeric mutant KT105/16S^NS11-3MnE^ (43.6 min) and comparable to that for KT105/16S^Eco^ (40.2 min). These data suggest that the deleterious effect of 16 S^NS11^ on the *E. coli* growth phenotype can be primarily explained by the base pair patterns formed between nucleotides 1416 and 1484.

## Discussion

In this study, we conducted a CRF analysis using the *E. coli* genetic system and investigated the functional evolvability of 16 S rRNAs, showing that the 16 S rRNA gene from a different phylum, Acidobacteria (16S^NS11^), whose sequence differed from the *E. coli* allele by 334 nucleotides, was functional in the *E. coli* ribosomal background. Further mutational analysis revealed that, among the 334 different sites, only a single base pair was deleterious, but the remaining 332 (99.4%) nucleotides were found to be similarly functional to those of *E. coli*. To the best of our knowledge, except for a preliminary study^29^ this is the first experimental study to report the similarity of 16 S rRNA genes between distantly related bacteria in terms of functionality, contradicting the notion of the complexity hypothesis that rRNAs coevolve with ribosomal proteins in a species-specific manner^10^.

Historically, comparative RNA sequence analysis of the 16 S rRNA^30^, which maps the variable nucleotides onto the secondary structure, revealed that nucleotides in this molecule generally co-vary so that mutations do not disrupt the secondary structure. Although there was no single confirmation that each species’ 16 S rRNA has equal functionality, such mutations were tentatively named compensatory neutral mutations^12, 13^, which, in turn, became a theoretical basis for the existence of neutral evolvability of the 16 S rRNA gene for use as a molecular clock for phylogenetic studies of prokaryotic species. In addition, the 16 S rRNA gene is regarded as a reliable phylogenetic marker because it has been assumed that the gene would rarely experience HGT, since the 16 S rRNA forms the structural core of the 30 S ribosome (complexity hypothesis)^1, 2^. On the basis of these backgrounds and the lack of fossil records for prokaryotes, 16 S rRNA was considered to be the *de facto* ultimate chronometer for the phylogenetic study of prokaryotes^3^. The current notion on the way microorganisms have evolved on Earth is based largely on the 16 S rRNA-based phylogenetic tree, which has been accepted widely over the last 30 years^3, 6^. However, the basic assumption—evolutionary neutrality of the 16 S rRNA gene—is based solely on comparative sequence analysis (and intuition) but not supported by any functional analysis. Structural and/or sequence comparison of naturally occurring ribosomes from different organisms are not necessarily informative because each individual ribosome (or any proteins/RNAs) has evolved in their evolutionary context, which should be rich in various biological noise^31^. It should especially be stressed that, apart from typical base-pairing nucleotides that intuitively seem to evolve following the mechanism of compensatory neutral mutations^12, 13^, it is unclear how mutations in free (unpaired) nucleotides, or those interacting with proteins, affect functionality of the ribosome; this is why this study aimed to rigorously investigate the *functional* evolvability of this molecule, which must be linked to the evolutionary history of this molecule in nature.

The results obtained through our CRF analysis led to the clear conclusion that bacterial 16 S rRNAs are significantly connected by a neutral network; the majority (99.4%,) of varying nucleotides (including free/unpaired and protein-binding nucleotides) that accumulated uniquely during the course of evolution of each organism (*E. coli* and acidobacterial species) were functionally similar (Figs 2 and 4), seemingly assuring that 16 S rRNA sequences can be used as a reasonable clock—at least in terms of neutral evolvability. Notably, this finding conversely suggests that the majority of critical interactions between 16 S rRNA and surrounding proteins *do not* evolve because of their stringent mutual constraints; slight sequence change can be extremely toxic. Establishment of such an inflexible framework or “cradle” must have been preceded by the divergence of 16 S rRNAs. More specifically, our results suggest that the common cradle for the 16 S rRNAs of Proteobacteria and Acidobacteria developed before the branching point of the two lineages and has remained unchanged. Since the divergence, 16 S rRNA genes of both lineages have accumulated mutations mainly by the compensatory neutral mutation mechanism, with relatively small number of stand-alone point mutations (Supplementary Fig. 1). This evolutionary model for the ribosome, which we call the cradle model, is in complete opposition to the complexity hypothesis^10^, which claims that the genes involved in complex biosystems, as represented by the ribosome, co-evolve with each other and thus scarcely experience HGT between species.

The finding that evolution of the 16 S rRNA gene may not follow the complexity hypothesis but instead follow the cradle model strongly suggest that HGT of 16 S rRNA genes between species (even across phyla) could have occurred more frequently than previously thought, with promiscuous nature representing an “inconvenient truth” for using the 16 S rRNA gene as a clock. Consistent with our cradle model and opposing the complexity hypothesis, ribosomal protein genes are reported to undergo frequent HGT^32, 33^. However, our results also suggest the presence of a minimal number of species-specific, non-neutral mutations in 16 S rRNA (e.g., single base pair incompatibility of nucleotides 1416 and 1484 [Figs 3 and 4]), which would work as a barrier for full-length transfer of the 16 S rRNA genes between species. A similar functional barrier was also observed between the 5′ and central domains (i.e., the pseudoknot in helix 2)^34^, which would prevent unfavorable HGT within two consecutive domains of the 16 S rRNA gene (Fig. 2). In other words, three consecutive domains from the 5′ end (i.e., the 5′, central, and 3′ major domains) or the 3′ major domain alone would be freely transferable between the 16 S rRNA genes of *E. coli* and Acidobacteria without a major perturbation in growth. Although the artificial chimera that we designed in this study was based on the well-defined domain structure of 16 S rRNA (Supplementary Fig. 1A and Supplementary Fig. 3), the transfer unit in nature may not encompass the full length of the gene, or a discrete domain, but can be a fragment of arbitrary lengths on a case-by-case basis. In fact, sporadic naturally occurring chimeric 16 S rRNA genes have been reported in a moderate number of bacterial species^35–41^, the recombination sites of which appear to be independent from domain-domain junctions. A systematic description of the chimeragenesis history of 16 S rRNA, which is technically difficult and has not yet been conducted, would be essential to estimate the evolutionary significance of the cradle model for the molecular evolution of 16 S rRNA genes.

In summary, the major experimental highlight of this paper is to provide a proof-of-concept that 16 S rRNA genes from two distantly related bacteria that differ at the phylum level (Acidobacteria and Proteobacteria in this case) are similar in function. This result, however, does not necessarily ensure that the same rule can be readily applied to other bacterial lineages. To further generalize our findings, the functionality of 16 S rRNA from other phylogenetic lineages or functionality of 16 S rRNAs using a different experimental setting needs to be tested (e.g., use of non-*E. coli* bacteria as a host). We anticipate that such systematic studies will help clarify Woese’s assumption that 16 S rRNA genes have universal neutral evolvability. Even if, however, experimental evidence shown in this and possible future studies suggest the universal neutral evolvability of 16 S rRNA genes and hence satisfies the necessary requirements to use the sequence as a logical molecular clock, this finding would simultaneously or inevitably imply the promiscuous nature of the 16 S rRNA gene, i.e., the occurrence of horizontal gene transfer among bacteria.

We are beginning to recognize that many genomes and genes have experienced HGT, which includes both operational and informational genes^10^, and the 16 S rRNA gene is no exception. It is undoubtedly true that organisms evolve following the tree-shape evolutionary model, but molecules or assemblages (i.e. genomes) thereof do not necessarily follow this model; in fact, they often violate it. Therefore, the evolutionary history of potentially promiscuous 16 S rRNA genes may well be described in a way that differs from the tree-shape. A more appropriate representation might be a network, web, or ring shape, which incorporates both vertical and horizontal evolutionary history. Discerning the vertical and horizontal information clearly and analyzing both would help elucidate how 16 S rRNAs and their hosts have evolved vertically and how the hosts interacted with each other upon molecular chimeragenesis.

## Materials and Methods

### Bacterial strains and growth conditions

*E. coli* KT101 (∆*rrnG* ∆*rrnA* ∆*rrnD* ∆*rrnE* ∆*rrnH* ∆*rrnB* ∆*rrnC*/pTRNA67, pRB101, *rna::Km* ^R^) is a derivative of SQ171 (∆7 prrn strain), a null mutant of the rRNA (*rrn*) operon in the chromosome^42–44^. The plasmid pRB101 (Amp^R^, *sacB*, pSC101 ori) contains the entire wild-type *rrnB* operon (including the 16 S rRNA gene), which complements the growth of KT101. The strain was cultivated at 37 °C in LB medium (1% [w/v] tryptone, 0.5% [w/v] yeast extract, 0.5% [w/v] NaCl) (Merck) containing 100 µg/mL ampicillin (Amp) and 25 µg/mL kanamycin (Km). *E. coli* KT105 is a derivative of KT101 in which pRB101 was completely replaced with pRB105 (*rrnB*, Tmp^R^, pSC101 ori) using sucrose-induced counterselection. The pRB105 plasmid was used as a vector for introducing foreign 16 S rRNA genes or mutagenesis experiments. The KT105 strain was cultivated in LB medium containing 25 µg/mL Km and 10 µg/mL trimethoprim (Tmp). The DTs of the KT105 derivative mutants were determined as described previously^11^. Briefly, 0.7 µL of overnight pre-culture was inoculated into 200 µL of LB/Km/Tmp in a flat-bottom 96-well plate (Corning). The plate was incubated at 37 °C with vigorous agitation (200 rpm) in a VersaMax plate reader (Molecular Devices) and the OD_600_ was monitored every 15 min for 24 h.

### Functional screening of foreign 16 S rRNA genes in *E. coli* ∆7

Functional screening of foreign (non-*E. coli*) 16 S rRNA genes from metagenome samples was carried out as described previously^11, 42, 45^, with slight modification^34^ (see Supplementary Materials and Methods and Supplementary Table 2). A total of ~4,000 colonies of KT105 were collected, from which 48 colonies were selected. Plasmid DNA (pRB105) was extracted from these mutants, and nucleotide sequences of the entire foreign 16 S rRNA genes were determined. A BLAST search^46^ was carried out using the National Center for Biotechnology Information (NCBI) nucleotide database, 16 S rRNA sequences (Bacteria and Archaea), with the program selection optimized for highly similar sequences (megablast). Sequence alignment of 16 S rRNA genes was performed using the SINA alignment service (http://www.arb-silva.de/aligner/).

### Mutagenesis

Domain-based chimeragenesis was carried out between the 16 S rRNA genes of *E. coli* and Acidobacteria as described previously^45^, with some modifications. For details, see Supplementary Materials and Methods and Supplementary Fig. 3. Site-specific point mutations were introduced to the 16 S rRNA gene using the QuikChange mutagenesis protocol^47^. Primer pairs used for mutagenesis experiments are listed in Supplementary Table 2.

### Data and materials availability

DNA sequence data for NS5 and NS11 16 S rRNA genes have been deposited under the accession numbers LC093165 and LC093166, respectively.

## Electronic supplementary material

Supplementary materials
