# Supplementary material for: Comparative RNA function analysis reveals high functional similarity between distantly related bacterial 16 S rRNAs
Source: Sci Rep. 2017 Aug 30;7:9993. doi: 10.1038/s41598-017-10214-3 (PMC5577257; doi:10.1038/s41598-017-10214-3)
Supplement: Supplementary file 1 — Supplementary materials [file 41598_2017_10214_MOESM1_ESM.pdf]

Supplementary Materials

Title

Full title: Comparative RNA function analysis reveals high functional similarity between distantly related bacterial 16S rRNAs

Authors

Miyuki Tsukuda,[1,2] Kei Kitahara,[3] Kentaro Miyazaki[1,2]*

Materials and Methods

PCR primer design for metagenomic library screening for functional 16S rRNAs in *E. coli* Δ7

Through genetic complementation assays of the *E. coli* KT101 (Δ7) strain [1-3], we previously showed that *E. coli* 16S rRNA ($16S^{Eco}$) can be replaced by foreign 16S rRNAs from diverse bacterial origins, including those from Gammaproteobacteria and Betaproteobacteria [4]. Therein, we amplified foreign (non-*E. coli*) 16S rRNA genes from environmental DNA samples using a set of universal primers, Bac8f and UN1541r, which are frequently used for phylogenetic and/or community analysis [5]. However, we noticed that these primers overlap variable sites, 19 (A or C) in Bac8f and 1527 (U or C) in UN1541r, respectively, and thus the amplicon could contain mutations. This was problematic, particularly for the former site, because nucleotide 19 pairs with nucleotide 916 to form a central pseudoknot structure, which is functionally critical [6-8], producing only modest results. Thus, we designed a new primer set, Bac1f and UN1542r (Supplementary Table 2), which encompassed nucleotides 1–18 and 1542–1528, respectively [9].

The PCR mixture contained 100 ng of template DNA (environmental metagenome), 1 × PCR buffer, 0.4 mM each of dNTPs, 0.25 μM each of primers (Bac1f and UN1542r), and 1 U of KOD FX-Neo DNA polymerase (Toyobo) in a total volume of 50 μL. The mixture was heated at 94°C for 2 min, then subjected to 30 rounds of thermal cycling at 98°C for 10 s, 48°C for 30 s, and 68°C for 1 min, followed by a final incubation at 68°C for 5 min. The amplicon was separated by agarose gel (0.8% [w/v]) electrophoresis; a single band was excised from the gel, purified, and dissolved in 30 μL of water.

An expression vector for the 16S rRNA gene was modified from pRB103 [4,10] by replacing the antibiotic selection marker from Zeocin to trimethoprim (Tmp) to yield pRB105, thereby avoiding undesired DNA fragmentation caused by Zeocin [11]. The entire vector (without the 16S rRNA gene) was PCR-amplified using the primer set Bac1r and UN1542f. The PCR mixture contained 1 × PCR buffer, 0.2 mM each of dNTPs, 1.5 mM $MgSO_4$, 0.25 μM of each primer, 10 ng of pRB105, and 1 U of KOD-Neo- DNA polymerase (Toyobo) in a total volume of 50 μL. The mixture was heated at 94°C for 2 min, then subjected to 25 cycles at 94°C for 10 s and 68°C for 5 min, followed by a final incubation at 68°C for 5 min. The products were treated with *Dpn*I (New England Biolabs, 10 U, 37°C, 6 h), gel-purified, and dissolved in 30 μL of water.

The 16S rRNA gene (~200 ng) and the linearized pRB105 (~200 ng) fragments were combined and ligated using the In-Fusion Cloning Kit (Clontech) in a total volume of 10 μL.

After incubation at 50°C for 1 h, the reaction products (2 μL) were introduced into competent *E. coli* JM109 cells (100 μL) and grown on LB/Tmp agar plates by incubating at 37°C overnight. The colonies were combined and the plasmids were extracted to yield a library. This plasmid library was then used to transform KT101. A functional screen based on the viability of the transformed KT101 was carried out as described previously [4], in which the selecting agent was changed from Zeocin to Tmp (10 μg/mL).

Domain-based chimeragenesis

Domain-based chimeragenesis was carried out between the 16S rRNA genes of Acidobacteria and *E. coli* using pRB105 carrying $16S^{NS11}$ as a template. Each domain (5′ [1–546], central [547–916], 3′ major [917–1388], and 3′ minor [1389–1542]; *E. coli* numbering) was PCR-amplified from the *E. coli* 16S rRNA (*rrsB*) gene using sets of the following primers: Bac1f and 5D– for the 5′ domain, CntD+ and CntD– for the central domain, 3MjD+ 3MjD– for the 3′ major domain, and 3MnD+ and UN1542r for the 3′minor domain (table S2, fig.. S3). For domain deletion in pRB105 carrying $16S^{NS11}$, pRB105 was amplified inversely using sets of the following primers: Bac1r and CntD+ for the 5′ domain deletion, 5D– and 3MjD+ for the central domain deletion, CntD– and 3MnD+ for the 3′major domain deletion, and 3MjD– and 1542f for the 3′minor domain deletion. PCR was performed using KOD Neo DNA polymerase (Toyobo) with the following temperature cycles: 94°C for 2 min, followed by 35 cycles of incubation at 94°C for 10 s, 48°C for 30 s, and 68°C for 15 s (for domain amplification) or 8 min (for domain deletion), and a final incubation at 68°C for 10 min. The amplicons were gel purified and dissolved in 30 μL of water. Each 16S rRNA domain fragment (~200 ng) and the cognate linearized vector fragment (~200 ng) were combined and ligated using the In-Fusion Cloning Kit (Clontech) in a total volume of 10 μL. After incubation at 50°C for 1 h, the products (2 μL) were introduced into competent *E. coli* JM109 cells. Correct shuffling products were confirmed by DNA sequencing and used for further studies.

***In vitro* translational activity**

The KT105 derivative strains were grown in 600 mL of LB/Km/Tmp in a 3 L flask at 37°C to OD_{600} 0.5–0.6. Cells were pelleted by centrifugation (5,000 *g*, 10 min, 4°C), resuspended in 10 mL of cold RBS-H buffer (20 mM HEPES-KOH [pH 7.6], 10 mM $Mg(OAc)_2$, 30 mM NH_4Cl, 6 mM 2-mercaptethanol), and transferred into 2 mL tubes (1.2 mL each). The tubes were centrifuged (36,220 *g*, 10 min, 4°C), supernatant was discarded, and the pellets were

combined with equal amount of glass beads (YGB05, Yasui Kikai) and 500 μL of RBS-H buffer. Cells were disrupted in a Taitec bead crusher (μT-12) at maximal speed for 1 min at room temperature, followed by chilling in an ice water bath for 2 min. This cycle was repeated three times to ensure complete disruption of the cells. The mixture was centrifuged at 15,000 rpm (36,220 *g*) for 10 min at 4°C. The supernatant was then transferred to a new tube.

The cell extract was then layered on top of a 10–40% (w/v) sucrose gradient prepared in RBS salt-wash buffer (20 mM HEPES-KOH [pH 7.6], 10 mM $Mg(OAc)_2$, 500 mM NH_4Cl, and 6 mM 2-mercaptoethanol) and separated by ultracentrifugation in a Beckman SW-40 Ti Rotor (35,000 rpm [245,853 *g*], 3 h, 4°C). Samples were taken from the top of the tube using a BIOCOMP Piston Gradient Fractionator, and the 70S ribosome was collected by monitoring at A_{254}. The 70S ribosome solution thus obtained (15 A_{254} each, ~400 A_{254} total) was overlaid onto an equal volume of 30% (w/v) sucrose in RBS-H buffer and ultracentrifuged at 40,000 rpm (280,976 *g*) for 12 h at 4°C in a Beckman SW-40 Ti rotor. The pellet was dissolved in RBS-L buffer (20 mM HEPES-KOH [pH 7.6], 6 mM $Mg(OAc)_2$, 30 mM KCl, 7 mM 2-mercaptoethanol) [12] to obtain pure ribosomes.

For translational activity measurements, the PUREfrex cell-free translational system (GeneFrontier) was used. The superfolder GFP [13,14] was used as a reporter; the gene was PCR-amplified, and the amplicon was gel-purified and dissolved in 30 μL of water. The reaction mixture (10 μL total) contained PUREfrex solution I (5 μL), solution II (0.5 μL), DNA template (20 ng), and 0.2 μmol of pure ribosomes. The reaction was monitored for an increase in GFP fluorescence (excitation: 488 nm; emission: 530 nm) for 1 min intervals at 37°C using a Stratagene Mx3000p qPCR system.

Sucrose density gradient analysis

Sucrose density gradient analysis of the ribosomes was carried out as previously described [1]. Briefly, KT105 derivatives were grown in 50 mL of LB/Km/Tmp in a 500 mL flask at 37°C. When the OD_{600} reached 0.4 to 0.6, 100 μg/mL chloramphenicol was added 5 min before harvesting to avoid polysome run off. The flask was rapidly chilled in ice water for 10 min, and the cells were collected by centrifugation (5,000 *g*, 10 min, 4°C). The pellets were resuspended in 1 mL of cold RBS-H buffer. Then, 15 μL of 0.5 mg/mL lysozyme (Sigma) was added, mixed and frozen at –80°C for 1 h. The sample was then incubated in ice water until completely thawed. This freeze-thaw cycle was repeated four times to ensure efficient recovery of the polysomes. The cell lysates were cleared by centrifugation (15,000 rpm

[36,220 *g*], 15 min, 4°C) and the supernatant was collected. Then, 5 units of the lysate at A_{260} were layered on top of a 10–40% (w/v) sucrose gradient prepared in RBS-H buffer, and separated by ultracentrifugation in a SW-40 Ti Rotor (35,000 rpm [245,853 *g*], 3 h, 4°C]. Samples were taken from the top of the tube using a BIOCOMP Piston Gradient Fractionator, and A_{254} was monitored continuously on an ATTO UV monitor (AC-5200).

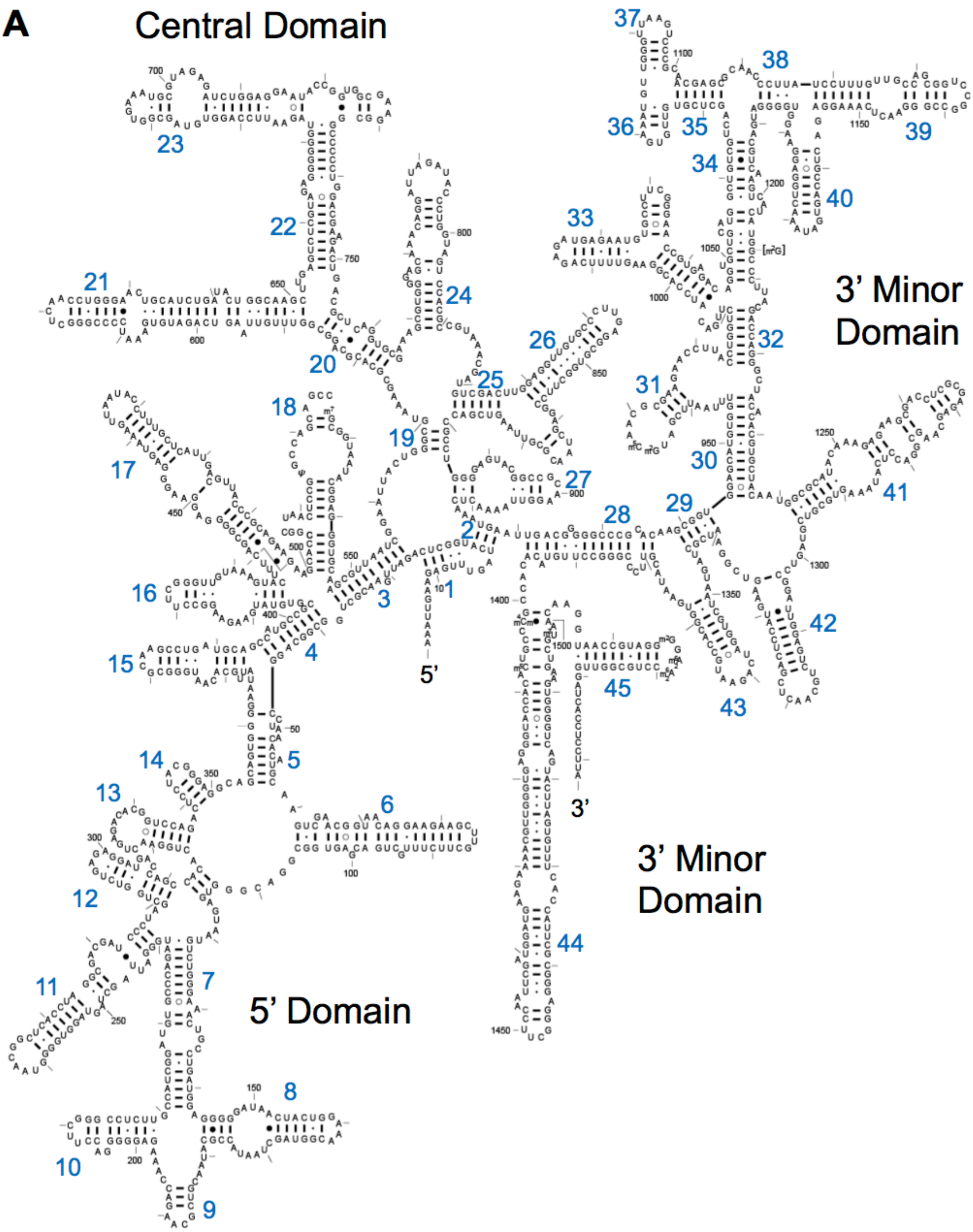
A
Central Domain
3' Minor Domain
3' Minor Domain
5' Domain
5'
3'
1
2
3
4
5
6
7
8
9
10
11
12
13
14
15
16
17
18
19
20
21
22
23
24
25
26
27
28
29
30
31
32
33
34
35
36
37
38
39
40
41
42
43
44
45

B

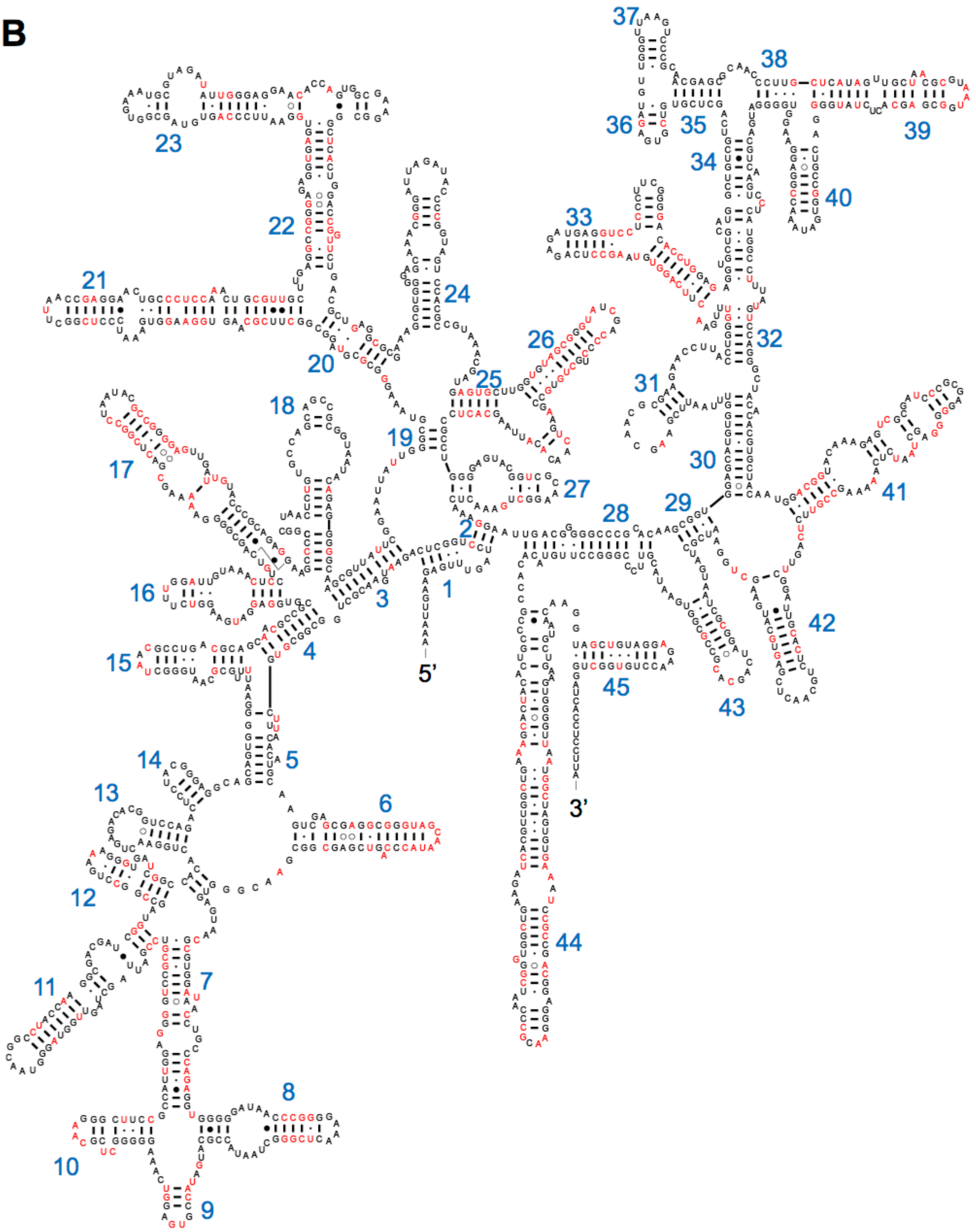

Supplementary Fig. 1. Secondary structure maps of 16S rRNAs

(**A**) *E. coli*. (**B**) NS11. The *E. coli* 16S rRNA map was obtained from the Comparative RNA website (http://www.rna.ccbb.utexas.edu/) [15]. The structure of NS11 was superimposed onto the *E. coli* secondary structure map; the nucleotides that are different from those of *E. coli* are shown in red in the NS11 structure.

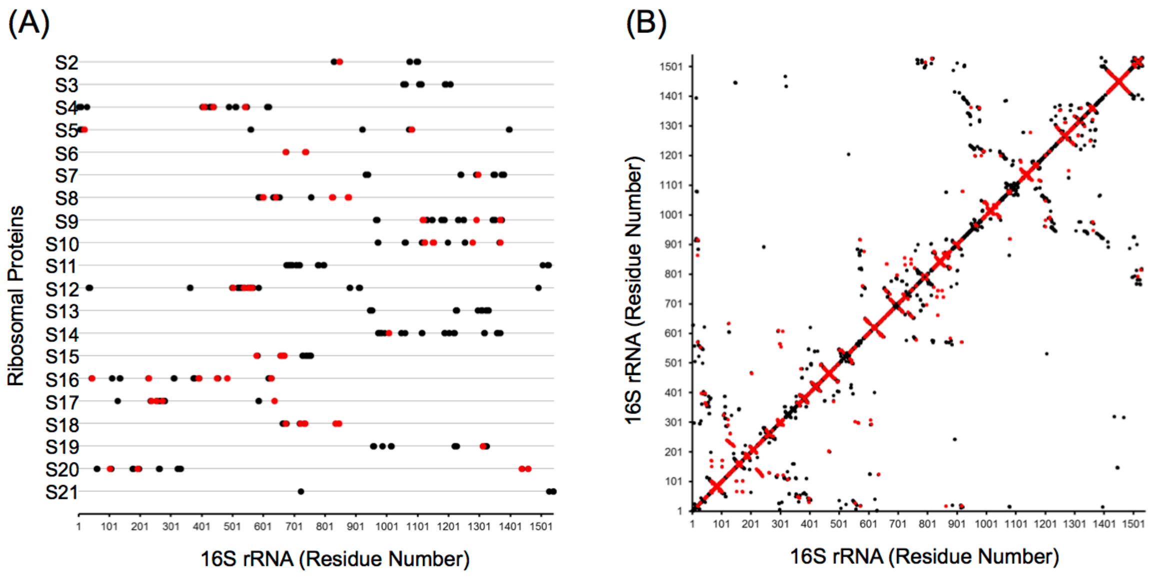

Supplementary Fig. 2. Analysis of atomic interactions between 16S rRNA and ribosomal proteins

Contact map showing interactions between (**A**) 16S rRNA－ribosomal proteins and (**B**) 16S rRNA－16S rRNA in the *E. coli* 30S subunit. Information on atomic interactions between oxygen, phosphorus, or nitrogen atoms within 3.4 Å distances were extracted from the crystal structure of the *E. coli* ribosome ribosome (PDB: 3R8O) [16] using CONTACT software [17] and dotted in black. Among them, contacts that involve mutated nucleotides in 16S^{NS11} are dotted in red. 16S rRNA contains 1542 nucleotides. S2-S21 denote the ribosomal proteins in 30S subunit (S1 is not included in the crystal structure). In the RNA–protein contact map (A), 88 of the total 410 RNA–protein interactions involved variable nucleotides. Thus, 73.7% nucleotides varied in NS11 do not interact with ribosomal proteins. Nucleotides 1416 and 1484 are not involved in interaction with proteins. It is known that genes for S9 and S17 can be knocked out in *E. coli* [18], suggesting that interactions between 16S rRNA and these proteins are also not essential. The other 18 ribosomal protein genes are reported to be essential [18]. In the RNA–RNA contact map (B), the interactions of the compensatory nucleotides within the RNA helices were seen as patterns that protruded perpendicular to the diagonal line (the nucleotide interaction between 1416 and 1484 is also seen in a protrusion). In this map, 285 nucleotides, representing 85.3% of the total 334 variable nucleotides, occurred in this protruding pattern, confirming the compensatory and conservative nature of the RNA secondary structure.

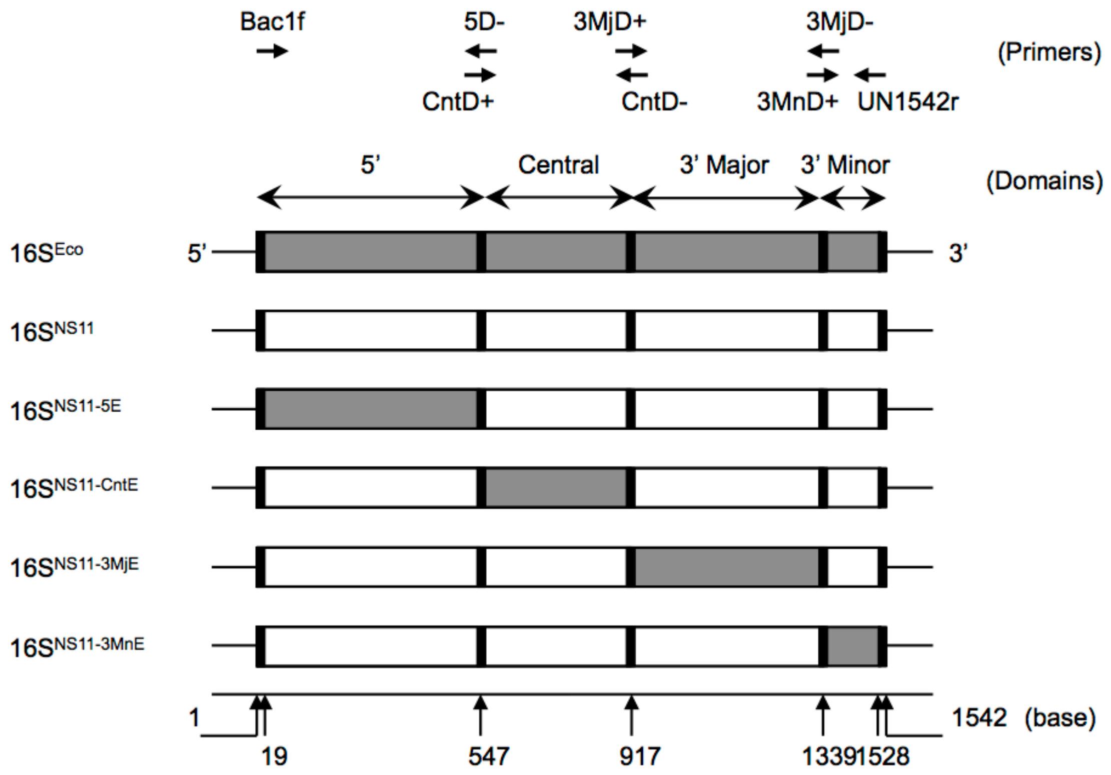

Supplementary Fig. 3. DNA constructs designed for domain-based chimeragenesis experiment

Schematic illustrations of DNA constructs ($16S^{Eco}$, $16S^{NS11}$, $16S^{NS11\text{-}5E}$, $16S^{NS11\text{-}CntE}$, $16S^{NS11\text{-}3MjE}$, and $16S^{NS11\text{-}3MnE}$) are shown. The $16S^{Eco}$ (full length; 1-1542) construct is colored in grey, $16S^{NS11}$ (19-1527) colored in white, and primer-binding sites are in black.

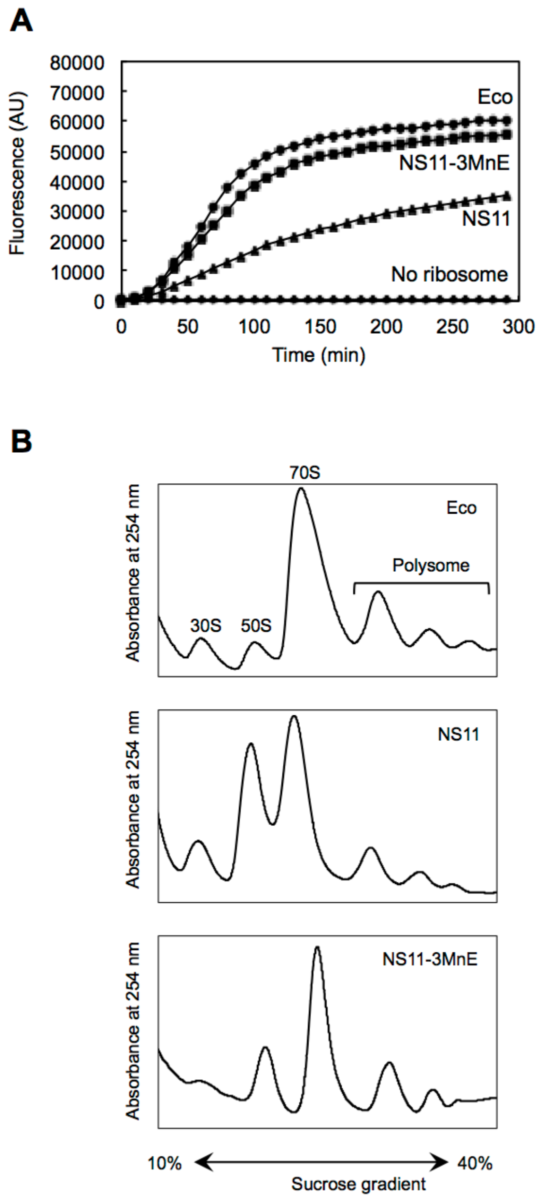

Supplementary Fig. 4. Biochemical characterization of mutant ribosomes

(**A**) *In vitro* translational activity of purified ribosomal samples containing *E. coli* (Eco), NS11, or NS11-3MnE 16S rRNAs. All ribosome samples were extracted from KT105 strains carrying the corresponding 16S rRNA genes. The ribosomal activity was determined using an *in vitro* transcription-translation system. The superfolder GFP [13,14] gene was used as a template. The reaction was carried out at 37°C, and fluorescence was measured at 10 min intervals. (**B**) Patterns of ribosomal subunit association. A sucrose density gradient analysis was conducted as described in the Materials and Methods.

Supplementary Table 1. Sequence information for acidobacterial 16S rRNA genes[1]

Clone	Accession number	Blast top hit (Accession number), identity	Phylogenetic classification	Identity to *E. coli rrsB*
NS5	LC093165	*Holophaga* sp. WY42 (KC921174.1), 89%	Acidobacteria	79.3%
NS11	LC093166	*Holophaga* sp. WY42 (KC921174.1), 88%	Acidobacteria	78.4%

[1]As a result of functional screening using KT101 (Δ7 prrn strain), we identified a total of 31 different 16S rRNA genes from diverse bacterial lineages, in which two genes belonging to phylum Acidobacteria were included. These genes, designated as $16S^{NS5}$ and $16S^{NS11}$, showed 79.3% ($16S^{NS5}$) and 78.4% ($16S^{NS11}$) identities, respectively, to the *E. coli* 16S rRNA gene ($16S^{Eco}$) and were 93.8% identical to each other.

Supplementary Table 2. Oligonucleotide primers used in this study

Primer	Sequence (5′ to 3′)
Bac1f	AAATTGAAGAGTTTGATC
UN1542r	TAAGGAGGTGATCCA
Bac1r	GATCAAACTCTTCAATTTAAAAGTTTGACGCTCAAAG
UN1542f	TGGATCACCTCCTTACCTTAAAGAAGCGT
5D-	CGCTTTACGCCCAGTAATTCCGATTAACGCT
CntD+	AGCGTTAATCGGAATTACTGGGCGTAAAGCG
CntD-	CCACCGCTTGTGCGGGCCCCCGTCAATTC
3MjD+	GAATTGACGGGGGCCCGCACAAGCGGTGG
3MjD-	CTCCCATGGTGTGACGGGCGGTGTGTACAAG
3MnD+	CTTGTACACACCGCCCGTCACACCATGGGAG

References

1 Kitahara, K. & Suzuki T. The ordered transcription of RNA domains is not essential for ribosome biogenesis in *Escherichia coli*. *Mol Cell* **34**:760-766, doi:10.1016/j.molcel.2009.05.014 (2009).

2 Asai, T., Zaporojets D., Squires C. & Squires C. L. An *Escherichia coli* strain with all chromosomal rRNA operons inactivated: complete exchange of rRNA genes between bacteria. *Proc Natl Acad Sci USA* **96**:1971-1976 (1999).

3 Quan, S., Skovgaard O., McLaughlin R. E., Buurman E. T. & Squires C. L. Markerless *Escherichia coli rrn* deletion strains for genetic determination of ribosomal binding sites. *G3* **5**:2555-2557, doi:10.1534/g3.115.022301 (2015).

4 Kitahara, K., Yasutake Y. & Miyazaki K. Mutational robustness of 16S ribosomal RNA, shown by experimental horizontal gene transfer in *Escherichia coli*. *Proc Natl Acad Sci USA* **109**:19220-19225, doi:10.1073/pnas.1213609109 (2012).

5 Weisburg, W. G., Barns S. M., Pelletier D. A. & Lane D. J. 16S ribosomal DNA amplification for phylogenetic study. *J Bacteriol* **173**:697-703 (1991).

6 Brink, M. F., Verbeet M. P. & de Boer H. A. Formation of the central pseudoknot in 16S rRNA is essential for initiation of translation. *EMBO J* **12**:3987-3996 (1993).

7 Dammel, C. S. & Noller H. F. A cold-sensitive mutation in 16S rRNA provides evidence for helical switching in ribosome assembly. *Genes Dev* **7**:660-670, doi:10.1101/gad.7.4.660 (1993).

8 Poot, R. A., van den Worm S. H., Pleij C. W. & van Duin J. Base complementarity in helix 2 of the central pseudoknot in 16S rRNA is essential for ribosome functioning. *Nucleic Acids Res* **26**:549-553 (1998).

9 Miyazaki, K., Sato M. & Tsukuda M. PCR primer design for 16S rRNAs for experimental horizontal gene transfer test in *Escherichia coli*. *Frontiers in Bioengineering and Biotechnology* **5**:14, doi:10.3389/fbioe.2017.00014 (2017).

10 Kitahara, K. & Miyazaki K. Specific inhibition of bacterial RNase T2 by helix 41 of 16S ribosomal RNA. *Nat Commun* **2**:549, doi:10.1038/ncomms1553 (2011).

11 Tsukuda, M. & Miyazaki K. DNA fragmentation caused by an overdose of Zeocin. *J Biosci Bioeng* **116**:644-646, doi:10.1016/j.jbiosc.2013.05.004 (2013).

12 Ohashi, H., Shimizu Y., Ying B. W. & Ueda T. Efficient protein selection based on ribosome display system with purified components. *Biochem Biophys Res Commun* **352**:270-276, doi:10.1016/j.bbrc.2006.11.017 (2007).

13 Pedelacq, J. D., Cabantous S., Tran T., Terwilliger T. C. & Waldo G. S. Engineering and characterization of a superfolder green fluorescent protein. *Nat Biotechnol* **24**:79-88, doi:10.1038/nbt1172 (2006).

14 Tsukuda, M. & Miyazaki K. Directed evolution study unveiling key sequence factors that affect translation efficiency in *Escherichia coli*. *J Biosci Bioeng* **116**:540-545, doi:10.1016/j.jbiosc.2013.05.013 (2013).

15 Cannone, J. J. *et al.* The comparative RNA web (CRW) site: an online database of comparative sequence and structure information for ribosomal, intron, and other RNAs. *BMC Bioinformatics* **3**:2 (2002).

16 Dunkle, J. A. *et al.* Structures of the bacterial ribosome in classical and hybrid states of tRNA binding. *Science* **332**:981-984, doi:10.1126/science.1202692 (2011).

17 The CCP4 suite: programs for protein crystallography. *Acta Crystallogr D Biol Crystallogr* **50**:760-763, doi:10.1107/s0907444994003112 (1994).

18 Shoji, S., Dambacher C. M., Shajani Z., Williamson J. R. & Schultz P. G. Systematic chromosomal deletion of bacterial ribosomal protein genes. *J Mol Biol* **413**:751-761, doi:10.1016/j.jmb.2011.09.004 (2011).
